# The Influence of Diffuse Nontoxic Goiter on the State of Protective Mechanisms of the Oral Cavity in Children

**DOI:** 10.25122/jml-2020-0013

**Published:** 2020

**Authors:** Oksana Ivanivna Godovantes, Tetiana Stepanivna Kitsak, Oleksandr Oleksandrovich Vitkovsky, Lyudmyla Vasylivna Kuzniak, Oleksii Serhiyovych Godovantes, Natalia Mykhaylivna Chaikovska, Larisa Yaroslavivna Fedoniuk

**Affiliations:** 1. Department of Pediatric Dentistry, Higher State Educational Establishment of Ukraine, “Bukovinian State Medical University”, Chernivtsi, Ukraine; 2. Department of Surgical Dentistry and Maxillofacial Surgery, Higher State Educational Establishment of Ukraine, “Bukovinian State Medical University”, Chernivtsi, Ukraine.; 3. Department of Pediatrics, Neonatology and Perinatal Medicine, Higher State Educational Establishment of Ukraine “Bukovinian State Medical University”, Chernivtsi, Ukraine; 4. Department of Foreign Languages, Higher State Educational Establishment of Ukraine, “Bukovinian State Medical University”, Chernivtsi, Ukraine; 5. Department of Medical Biology, I. Horbachevsky Ternopil National Medical University, Ternopil, Ukraine

**Keywords:** children, diffuse nontoxic goiter, dental pathology, local protective mechanisms

## Abstract

Immunopathogenesis of inflammatory and dystrophic diseases of the tissues of the oral cavity is characterized by cellular and humoral factors of specific and nonspecific resistance, the functioning of which is determined by the overall somatic state.

This study aimed to study the features of protective mechanisms of the oral cavity due to orthodontic pathology, pathology of periodontal tissues, and odontogenic inflammatory process in children with diffuse nontoxic goiter.

Eighty children with diffuse nontoxic goiter aged 12-15 years with different dental status were examined. Evaluation of local immunity of the oral cavity was carried out by determining the content of sIgA, IgA, IgG, lysozyme activity, and levels of IL-1β, IL-4 by enzyme immunoassay.

Immunological studies have shown that in children with diffuse nontoxic goiter, the activity of lysozyme in the oral fluid is decreased. The level of sIgА is also reduced by about 20%. Besides, there is an increase in the content of IgG and a growing trend in the level of IgА. The content of IL-1β and IL-4 in such children fluctuates more compared to somatically healthy children.

In conclusion, a violation of the local protective mechanisms of the oral cavity is observed in children with diffuse nontoxic goiter. Also, the increase in the severity of dental pathology leads to increased tension of local protective and compensatory reactions.

## Introduction

The local protective mechanisms of the oral cavity are known to play an essential role in the development of dental diseases [1-3]. At the same time, they are sensitive markers of the general state of the child's body, through which the pathological influence of somatic diseases on the hard and soft tissues of the dentition is often realized.

The formation of a local protective barrier is based on the interaction of mucosal-secretory, phagocytic-cellular, humoral, and immunoregulatory systems [4-6].

The secretion of the salivary glands acts as a protective barrier, preventing bacteria from attaching to epithelial cells. It does not only wash away microorganisms but also has a bactericidal activity due to the presence of biologically active substances in it. The humoral factors of natural protection include the mucolytic enzyme lysozyme.

Lysozyme, as a hydrolytic enzyme, is able to break down specific polysaccharides of bacterial cell walls. As a cationic protein, having an affinity to negatively charged areas of cell membranes of tissues, it has a wide range of physiological effects: bacteriolytic, bacteriostatic, immunomodulatory, regulatory, and others [7].

The function of specific humoral immunity in protection against extracellular pathogens is carried out by antigen-specific antibodies, which are synthesized by plasma cells and enter the oral cavity through the secretion of salivary glands and dentogingival connection. The largest is the content of secretory immunoglobulin (sIgA); the levels of IgA and IgG are also quite high. The participation of antibodies in the immune defense is realized in the following directions: neutralization of the pathogen and its toxins (reaction of antibody-dependent cytotoxicity), activation of complement, and opsonization. The main effectors are neutrophils, which provide phagocytosis of microorganisms. Their absorptive and bactericidal ability is sharply enhanced in the presence of complement and IgG [8].

The key point in the development of the pathological process with the participation of immunocompetent cells is represented by the inflammatory mediators. The initiator of the cytokine cascade is interleukin 1β (IL-1β), produced by the cells of the body in response to the action of microbes and their toxins, inflammatory agents, activated complement components. It has the ability to stimulate T- and B-lymphocytes, increase the production of other cytokines, enhance chemotaxis, phagocytosis, hematopoiesis, vascular permeability, cytotoxic, and bactericidal activity [9-10].

In response to the increased production of proinflammatory cytokines, T-cells and monocytes produce cytokines with a pronounced anti-inflammatory effect, typical of which is IL-4. It can suppress certain parts of the inflammatory response, participates in the humoral component of the immune response: stimulation of immunoglobulin secretion in lymphocytes.

Thus, the immunopathogenesis of inflammatory and dystrophic diseases of the tissues of the oral cavity is realized through cellular and humoral factors of specific and nonspecific resistance, the functioning of which is determined by the overall somatic state of the child's body.

The work aimed to study the features of protective mechanisms of the oral cavity due to orthodontic pathology, pathology of periodontal tissues, and odontogenic inflammatory process in children with diffuse nontoxic goiter (DNG).

## Material and Methods

To achieve this goal, 80 children with DNG, aged 12-15 years, have been examined. Observation groups were formed as follows: group I -children with good dental health (n=25); group II - children with chronic catarrhal gingivitis (CCG) (n=25); group III - children with odontogenic inflammatory processes (n=15); group IV - children with orthodontic pathology (n=15). The control groups were somatically healthy children with similar dental diseases of the same age: IC group - (n=25); IIC group - (n=25); IIIK group - (n=25); IVK group - (n=25).

The evaluation of local immunity of the oral cavity was carried out by determining the content of sIgA, IgA, IgG by simple radial diffusion technique in agar according to G. Manchini (1963) using monospecific standard antisera against the studied classes of immunoglobulins, lysozyme activity by G. Gorin's method (1971) in modification by A. P. Levitskyi and O.O. Zhygina (1974) and levels of IL-1β, IL-4 by enzyme immunoassay.

The oral fluid was collected in the morning after rinsing the mouth with distilled water by spitting into test tubes. The samples with oral fluid were centrifuged at 3000 × g for 15 minutes, and the supernatant was used. The samples were stored frozen at -20°C. A calibration curve was formed based on the optical density of the standards to calculate the obtained cytokine concentration.

The obtained results were processed statistically using Student's t-test.

## Results and Discussions

Immunological studies have shown that in children with DNG, there is a decrease in the activity of lysozyme in the oral fluid, both in the study of dental pathology and under the conditions of healthy oral tissues (Table 1). The highest level of lysozyme in the oral fluid of children on the background of DNG is in the case of orthodontic pathology (30.27 ±1.84) u/l. In the case of CCG and odontogenic inflammatory processes, a significant decrease in the level of lysozyme in the oral fluid is observed. The lowest enzyme activity was recorded in children with odontogenic inflammatory processes (18.45±1.07) u/l.

**Table 1:** The level of lysozyme activity in the oral fluid of children in observation groups, depending on the somatic and dental condition, u/l (M±m).

Groups of children	Children with diffuse nontoxic goiter	Somatically healthy children
**Dental healthy children**	29.71 ± 1.54	34.56 ± 2.13
**Children with orthodontic pathology**	30.27 ± 1.84	34.21 ± 1.75
**Children with chronic catarrhal gingivitis**	26.69 ± 1.16	32.01 ± 0.15
**Children with odontogenic inflammatory processes**	18.45 ± 1.07	25.83 ± 1.12

**Figure 1:**
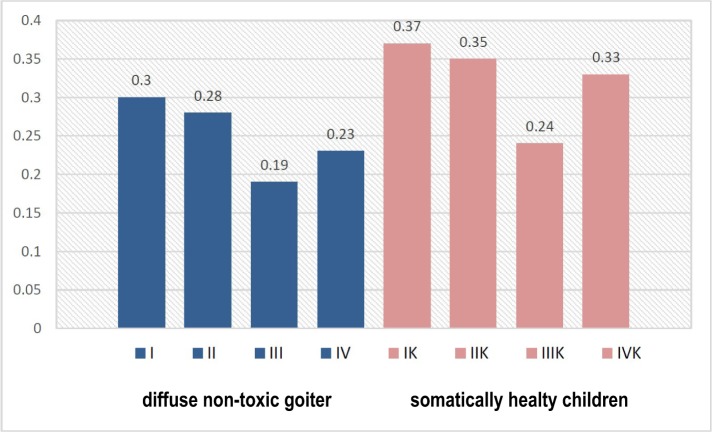
The level of sIgA in the oral fluid of children in observation groups. depending on the somatic and dental condition (g/l).

**Figure 2:**
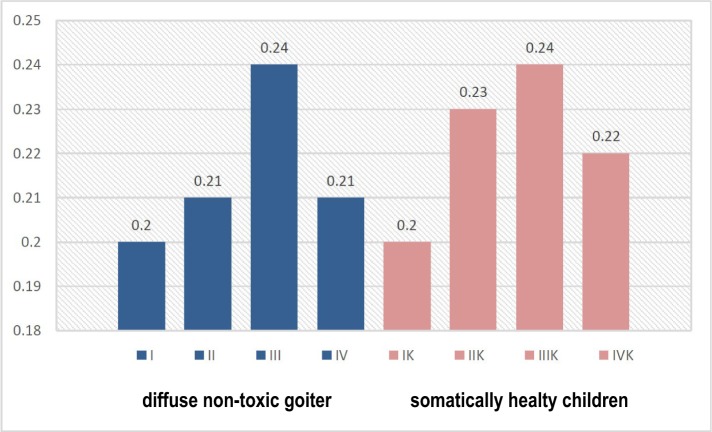
The level of IgA in the oral fluid of children in observation groups, depending on the somatic and dental condition (g/l).

**Figure 3:**
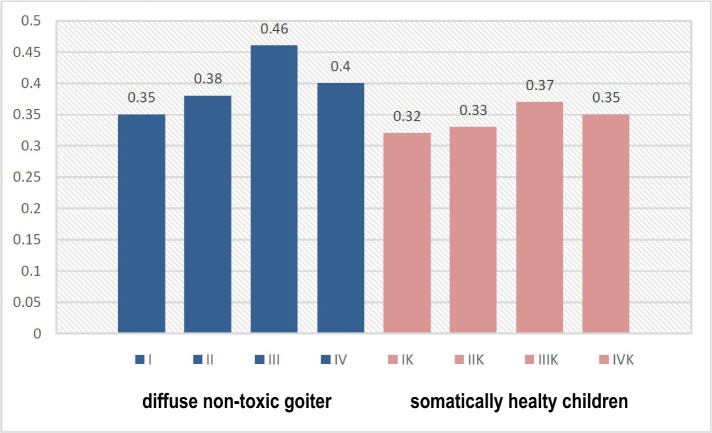
The level of IgG in the oral fluid of children in observation groups, depending on the somatic and dental condition (g/l).

According to the content of immunoglobulins, we found a clear pattern of reduced levels of sIgА and increased levels of IgА and IgG with the deterioration of the dental status of children (Figures 1-3). This was especially evident in the conditions of DNG, which indicates the influence of somatic pathology on the functioning of the protective mechanisms of the oral cavity.

In particular, in children of group I, the content of sIgA in the oral fluid was 18.9%, lower compared to the figures of the control group; in groups II, III and IV – the value was approximately 20%. We found out an increase in the level of sIgА in children with mild and moderate CCG, which can be explained by the protective-compensatory mechanism of a specific immune response.

The increase in the content of IgG (р<0.05), along with an increasing tendency of the IgА levels, indicate an increased intensity of local immunity of the oral cavity in children with DNG.

**Figure 4:**
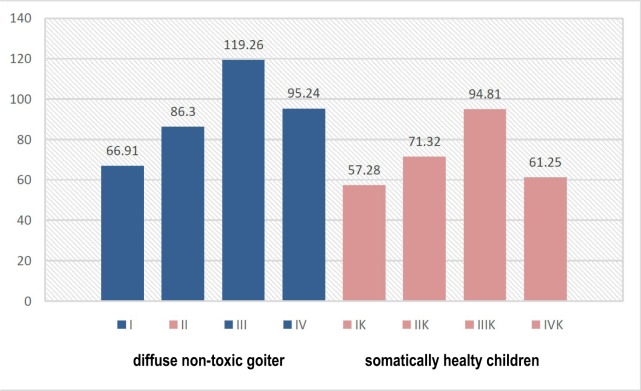
The level of IL-1β in the oral fluid of children in observation groups, depending on the somatic and dental condition (pg/ml).

**Figure 5:**
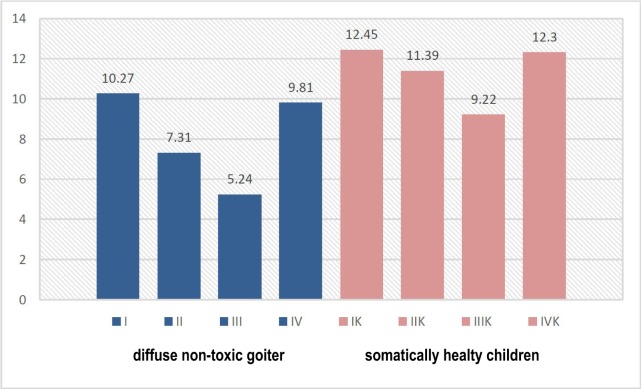
The level of IL-4 in the oral fluid of children in observation groups, depending on the somatic and dental condition (pg/ml).

The detection of cytokines in the oral fluid of children in the observation groups showed the opposite dynamics of changes for both mediators. The content of IL-1β and IL-4 in children with DNG fluctuated more compared to somatically healthy children (Figures 4, 5).

## Conclusions

A violation of the local protective mechanisms of the oral cavity is observed in children with DNG, which is characterized by a decrease in the sIgА levels, an increase in the content of IgА and IgG, a drop in lysozyme activity and an imbalance of pro-and anti-inflammatory cytokines. Also, the increase in the severity of dental pathology leads to increased tension of local protective and compensatory reactions.

The prospect of further research is to carry out targeted, pathogenetically justified correction of violations of local immunity in children during dental treatments.

## Conflict of Interest

The authors confirm that there are no conflicts of interest.
